# Current News

**Published:** 1889-12

**Authors:** 


### Current News.

### CHICAGO AND VICINITY.

The dentists of Chicago have subscribed for more than fifteen thousand dollars of the Stock of the World's Fair.

Dr. H. J. McKellops, of St. Louis, was the guest of the Odontological Society, at their recent meeting, held November 19, at the Leland Hotel.

Dr. E. S. Talbot is delivering a special course of lectures at the University Dental College, upon the etiology of the Irregularities of the Teeth. These lectures will shortly appear as a portion of the second edition of his book on "Irregularities of the Teeth."

The dental colleges of Chicago have the largest classes this session of any year in their history. This is doubtless due to the recent action of the National Association of Dental Faculties, making it obligatory upon all colleges of the Association to require, after the session of 1790–91, three years of college instruction of all students before graduation.

Dr. Truman W. Brophy, Dean of the Chicago College of Dental Surgery, has been suffering from a severe attack of bronchitis and has gone to California for the winter. His many friends will hope to hear of his speedy recovery.

At the October meeting of the Chicago Dental Club it was voted to make the International Dental Journal the official organ of that body, and hereafter it will publish its proceedings in that Journal.

Dr. Crouse is not growing thin over the suit for damages brought against him by the International Tooth Crown Company for his efforts in protecting the dentists against the claims of this company. If you are not already a member of the Protective Association, it would be wise to get in as soon as possible.

The first time you are in Chicago call on Dr. R. F. Ludwig and see his new suspension engine, run by the electric motor. It is novel and quite inexpensive, and we hope he will place it on the market.

---

Dr. John S. Marshall has been appointed secretary of the National Association of Dental Faculties *vice* Dr. J. E. Cravens, who has resigned on account of his removal to Paris, France.

---

The idea of a grand "memorial meeting" of the American Dental Association, in 1892, pleases the dentists in this neighborhood, and if the World's Fair should be located in Chicago, the profession at home and abroad will receive a royal welcome.

---

Some members of the profession put down their names as members of the Dental Protective Association of America, agreeing to pay up on or before January 1, 1890. Dr. Crouse desires to remind them that the day is near at hand.

---

Dental Protective Association.—At a regular meeting, held October 28, 1889, it was resolved, that the Brooklyn Dental Society endorse the Dental Protective Association and recommend its members to join said association at once. Louis Shaw,

*Corresponding Secretary.*

Brooklyn, November 15, 1889.

---

At the Georgia State meeting several members reported cases of teeth assuming the color of green glass, apparently due to the use of the "Howe screw-post." It was found very difficult, if not impossible, to bleach the tooth and restore the natural color.

---

Something must have come over the spirit of the dreams of the editor of the *Cosmos* in that two rather stale jokes have crept into the editorial columns of that staid journal lately.

---

Rubber dam should be thoroughly washed in soap and water, perfumed, and kept in an air-tight case. Place a very small, fine napkin between the lips and the dam. B. H. Catching.

### NATIONAL ASSOCIATION OF DENTAL FACULTIES.

*Special Notice.*

The resignation and removal to Paris, France, of Dr. Junius E. Cravens, has made necessary the appointment of a secretary to this position.

On the recommendation of the Executive Committee, I have appointed Dr. John S. Marshall, of Chicago. All communications requiring the attention of this organization should hereafter be sent to his address, No. 9 Jackson Street, Chicago, Ill.

JAMES TRUMAN,
*President N. A. of D. F.*

PHILADELPHIA, November 25, 1889.

---

AT the annual meeting of the New England Dental Society, held in Springfield, Mass., October 23, 24, 25, the following were elected officers for the ensuing year: President, Dr. C. W. Clement, Manchester, N. H.; First Vice-President, Dr. W. E. Page, Boston, Mass.; Second Vice-President, Dr. J. F. Adams, Worcester, Mass.; Secretary, Dr. Edgar O. Kinsman, Cambridge, Mass.; Assistant Secretary, Dr. W. P. Cooke, Boston, Mass.; Treasurer, Dr. G. A. Gerry, Lowell, Mass.; Librarian, Dr. A. H. Gilson, Boston, Mass.

*Executive Committee.*—Dr. C. A. Brackett, Newport, R. I.; Dr. S. G. Stevens, Boston, Mass.; Dr. J. H. McQuade, Medford, Mass.; Dr. George F. Cheney, St. Johnsbury, Vt.; Dr. George A. Young, Concord, N. H.

EDGAR O. KINSMAN, D.D.S.,
*Secretary.*

CAMBRIDGE, MASS.

---

AT the annual meeting of the Odontological Society of Chicago, held November 19, the following officers were elected for the ensuing year: President, Dr. P. J. Kester; Vice-President, Dr. F. H. Gardiner; Secretary and Treasurer, Dr. E. Noyes; Curator, Dr. Garrett Newkirk, New York; member of Board of Censors, Dr. George H. Cushing.

E. NOYES,
*Secretary.*

CHICAGO, November 27, 1889.

